# Psychological well-being and death anxiety among breast cancer survivors during the Covid-19 pandemic: the mediating role of self-compassion

**DOI:** 10.1186/s12905-021-01533-9

**Published:** 2021-11-03

**Authors:** Majid Yousefi Afrashteh, Samin Masoumi

**Affiliations:** Department of Psychology, Faculty of Humanities, University of Zanjan, Zanjan, Iran

**Keywords:** Psychological well-being, Death anxiety, Breast cancer, Self-compassion, COVID-19

## Abstract

**Background:**

Despite the abundance of clinical data available for Coronavirus Disease 2019 (COVID-19), little research on the psychological well-being of breast cancer survivors has been published. We investigate the extent to which self-compassion accounted for the association between psychological well-being (depression, anxiety) and death anxiety in breast cancer survivors.

**Methods:**

A cross-sectional study design was applied. Participants were recruited from three departments of oncology in Zanjan, Iran. Data were collected from 210 breast cancer patients. Participants completed self-report measures. Pearson correlation coefficient was used to assess the relationship among the study variables. Bootstrapping analyses were used to test the significance of indirect effects.

**Results:**

Correlational analyses revealed that depression and anxiety were significantly and positively related to death anxiety (*r* = 0.77, *p* < 0.01; *r* = 0.85, *p* < 0.01, respectively) and negatively to self-compassion (*r* = − 0.48, *p* < 0.01; *r* = − 0.53, *p* < 0.01, respectively). Bootstrapping analyses revealed significant indirect effects of depression (*β* = 0.065, *SE *= 0.35, *p* < 0.03, 95% *CI* [*LL *= − 0.0083, *UL*: − 0.1654]) and anxiety (*β* = 0.089, *SE *= 0.09, *p* < 0.04, 95% *CI* [*LL *= − 0.0247, *UL*: − 0.1987]) on death anxiety through self-compassion.

**Conclusions:**

Findings from this study indicate that self-compassion may be considered as one treatment strategy to improve psychological well-being of cancer patients in the new context of the COVID-19 pandemic.

## Introduction

It is universally accepted that the first confirmed COVID-19 case was recognized in Wuhan, China [1]. Coronavirus is a contagious disease [2], that has been rapidly spreading in almost all countries of the world from South East Asia to Central Europe [3]. On February18, 2020, the first recorded COVID-19 case was officially declared in Iran, when a real-time polymerase chain reaction (PCR) analysis of two cases who died in Qom turned positive for COVID-19 [4]. The results of the current COVID-19 pandemic such as severe acute respiratory syndrome and high mortality [5], the significant changes caused by social distancing in behavior associated with the COVID-19 pandemic, may exclusively and completely have noticeable effects on mental health [6, 7] especially in populations with serious illness.

The COVID-19 pandemic may increase concerns among breast cancer survivors about the severe risks of contracting COVID-19 in clinical settings, as well as fears linked to previous stressful health experiences (for instance, being hospitalized during care and also being isolated due to neutropenia) [8] prompting them to delay treatment. Severe anxiety and loneliness brought on by social isolation and quarantine, particularly when applied for long time, are linked to a higher risk of death in people with cancer [9]. Death anxiety may be exacerbated by the epidemic’s unpredictability over death [10]. Death anxiety is characterized as a feeling of concern, fear, or dread brought about by the realistic prospect of dying, as opposed to general anxiety [11]. Fear of death disrupts a cancer patient’s adaptive process and destroys their future dreams [12]. Taking into consideration the negative effects of death anxiety, several researchers have begun to investigate the determinants of death anxiety [13, 14].

The devastating effect of COVID-19 on mental health could be much more serious than normally expected [15]. Despite the fact that the long term consequences of a disease epidemic on mental health are expensive, the effect of a global pandemic on mental health is often overlooked during COVID-19 pandemic control [16]. Regarding the current situation, a recent study and meta-analysis of the pandemic’s effect on mental health, based on 13 studies conducted exclusively in Asian countries, reported that depression and anxiety are often more than 20% widespread [17]. During the COVID-19 pandemic, anxiety and depression in cancer sufferers can be increased through quarantine and physical distancing techniques and keep the population down, particularly when used for extended periods of time [18]. Because of their immunocompromised process characterized by both cancer and multiple anticancer therapies, oncology patients are treated as a vulnerable group during COVID-19 pandemic, with a remarkably high mortality rate [19]. These patients have a variety of issues, including pain, sleeplessness, physical exhaustion, and a low quality of life as consequences of extreme anxiety and depression [20]. Due to these psychological aspects, individuals with a terminal condition, such as cancer, suffer greater death anxiety than those with other serious illnesses [21].

People may blame not only themselves but also others for not fulfilling their own and their families’ needs in light of the current disease outbreak [22, 23]. Mental health providers apply psychological strategies including compassion and respect for people at this crucial moment [24, 25]. A relatively new dispositional approach to psychopathology is self-compassion, which describes how directing compassion toward the self can provide a buffer against adversity [26]. Relatively high levels of self-compassion have been shown to delay or mitigate symptoms of anxiety and depression [27, 28]. An analysis of cancer patients found that having more self-compassion is linked to experiencing less psychopathological problems and getting a better quality of life [29]. According to a study by Proeve et al. high levels of self-compassion can prevent or reduce the onset of anxiety and depression [28].

## Present research

**Fig. 1:**
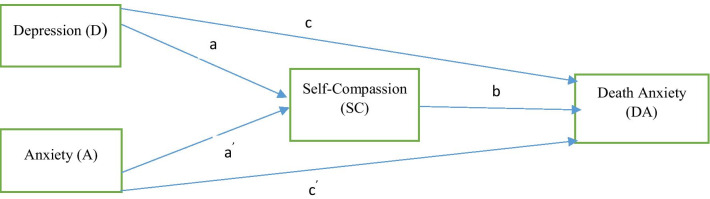
A conceptual diagram of the hypothesized mediation model, including. a, a′, the effects of the independent variables D and A on mediator SC; c, c′ the direct effects of D and A on dependent variable DA; b the effect of the mediator SC on dependent variable DA

Currently there is little data on the effect of COVID-19 on cancer sufferers. As a consequence, it is important to understand the psychological impact of the COVID-19 pandemic on breast cancer patients, such as fear of death, anxiety, and depression. The purpose of this research is to investigate the relationship between psychological wellbeing (anxiety and depression), self-compassion and death anxiety in breast cancer survivors during the COVID-19 pandemic. Therefore, the first objective of this study was to examine the relationship between depression, anxiety, and self-compassion with death anxiety. The second objective was to examine the mediating effects of self-compassion on these associations. Figure 1 shows the conceptual diagram of the research.

## Method

### Participants

The participants were recruited from three oncology departments located in Zanjan, Iran. Recruitment occurred from September 26th to November 15th 2020. During this time, 210 patients showed interest to participate in the study and were selected through non-random (convenient sampling). All study participants were female with breast cancer. Participants had mean age of 38.97 (SD = 12.37) and ranging between 18 and 65. According to adequate power for Structural Equation Modeling (SEM), this was 5% more than the suggested sample size [30] and this indicates that sample size is big enough to test our hypothesis. Demographics of patients are shown in Table 1.

**Table 1:** Demographic and treatment characteristics of breast cancer patients (N = 210)

Characteristics	Mean (SD)	N (%)
*Age*	38.97 (12.37)	
18–30		48 (22.90)
31–40		69 (32.9)
41–50		55 (26.2)
51–65		38 (18.1)
*Education*		
Less than diploma		19 (9)
Diploma*		54 (25.7)
Associate’s degree		73 (34.8)
Bachelor’s degree		39 (18.6)
Master’s and higher		25 (11.9)
*Marital status*		
Single		98 (46.7)
Married		112 (53.3)
*Type of therapy*		
Lumpectomy + chemotherapy		41 (19.5)
Mastectomy + chemotherapy		74 (35.2)
Chemotherapy		57 (27.1)
Radiotherapy		38 (18.1)

*High school

### Procedure

This is a cross-sectional study. Patients had already been diagnosed with breast cancer in hospitals and were able to individually complete the questionnaires. With permission from clinician, participants were invited by the corresponding author during their doctor’s appointment to complete a number of self-report questionnaires. The main inclusion criteria were: (1) attended their doctor’s appointment regularly (2) survivors haven’t received any psychological interventions (3) be able to understand and read Farsi (4) have received at least one or two of these treatments (Chemotherapy, Radiotherapy, Mastectomy and Lumpectomy). Among the 385 patients approached in the hospitals, 210 patients showed interest to participate in the study, 87 did not want to be included in the study, 75 were not included because of the excluding criterion and 13 of them did not fill in the forms. The response rate was finally 0.54. Participants who completed the questionnaires less than 5 min or more than 20 min were excluded. All rights of the participants were protected during this study. All procedures performed in study involving human participants were according to the ethical standards of the National Research Committee. This study was approved by research committee of University of Zanjan. Individuals completed a consent document prior to survey and they had the right to leave the study at any time.

### Instruments

#### Demographics collection sheet

Patients were requested to provide the following demographic data (age, level of education, marital status) and data related to cancer treatment received at the time of diagnosis.

#### Templer’s death anxiety scale (DAS)

Templer’s death anxiety scale contains 15 questions that assess the subjects’ attitudes towards death, such as “I am very much afraid to die”, “I fear dying a painful death”, and “The sight of a dead body is horrifying to me”. The subject answer to each question with a “yes” or “no”, where “yes” indicates anxiety in that area. Total scores range from 0 to 15, with a higher score corresponding to a higher level of anxiety [31]. Templer obtained a test–retest reliability coefficient of 0.83 for the scale [31]. The DAS has already been translated and widely used in Iran.

#### Beck depression inventory-II (BDI-II [32])

To measure depressive symptoms within the past 2 weeks, the 21-item BDI-II was used. For each item, participants were asked to indicate the statement that best described the way they felt during the past 2 weeks, including the day of scale administration. For example, participants were asked to indicate how often they felt sadness from the following options: 0 (“I do not feel sad”), 1 (“I feel sad much of the time”), 2 (“I am sad all of the time”), or 3 (“I am so sad or unhappy that I can’t stand it”). Items were scored on a scale from 0 to 3. A composite score was created by summing the items, such that higher scores indicated more depressive symptoms. The BDI-II has already been translated in Iran and is widely used.

#### Beck anxiety inventory (BAI [33])

The 21-item BAI was used to assess participants’ experiences of symptoms related to anxiety within the past month. Items (e.g., ‘fear of worst happening,’ ‘hands trembling’) were rated on a scale from 0 (not at all) to 3 (severely, it bothered me a lot). A composite score was created by summing the 21 items, such that larger scores correlate with more anxiety. The BAI has already been translated and widely used in Iran.

#### Self-compassion scale (SCS)

The 26-item SC scale was used to assess how participants typically act towards themselves in difficult time [34]. On a scale from 1 (almost never) to 5 (almost always), participants rated the frequency with which they behave in the manner stated in each statement (e.g., ‘When times are really difficult, I tend to be tough on myself’). To compute a composite score, the negative items were reversed scored, and the sum across all items was computed. Higher scores indicated greater self-compassion. The SCS has already been translated in Iran and is widely used.

### Statistical analysis

Statistical analyses for this study were performed using IBM SPSS Statistics software 23 (IBM SPSS Statistics for Mac, 2015). Descriptive statistics were computed for demographic and medical characteristics. To assess for mediation, a series of correlations were first conducted to investigate associations between variables. Pearson correlations were used to test bivariate associations of death anxiety, well-being (depression and anxiety) and self-compassion. Then, a series of path analyses were run to investigate if self-compassion mediated the relationship between psychological well-being (depression and anxiety) and death anxiety. Path analyses were performed using AMOS 23 with maximum likelihood estimation. In this study, bias-corrected bootstrap confidence intervals for indirect effects were based on 210 samples and were considered significant if they did not include 0 [35]. Bootstrapping analyses revealed significant indirect effects of psychological well-being (depression and anxiety) on death anxiety via self-compassion. Bootstrapping is considered to be superior to alternative tests of indirect effects [36, 37]. A significance criterion of *p* < 0.05 was used for all statistical analyses.

## Results

We conducted correlation analyses to investigate the relationships among all the variables. Means, SDs, minimum, maximum, range scores and Cronbach‘s alpha values of variables are indicated in Table 2. The mean score of depression was 58.5 (SD = 4.30), anxiety 58.1 (SD = 3.62), self-compassion 99.5 (SD = 20.7), and death anxiety 14.3 (SD = 1.1).

**Table 2:** Descriptive statistics for research variables

Variables	Mean	SD	Range	Minimum	Maximum	α
Depression	58.5	4.30	15	48	63	0.85
Anxiety	58.1	3.62	13	50	63	0.89
Self-compassion	99.5	20.7	52	78	130	0.90
Death anxiety	14.3	1.1	4	12	15	0.78

Both depression and anxiety were significantly and positively related to death anxiety and negatively related to self-compassion. Table 3 showed a negative relationship between self-compassion with death anxiety (*r* = − 0.46, *p* < 0.01), self-compassion with depression (*r *= − 0.48, *p* < 0.01), and self-compassion with anxiety (*r* = − 0.53, *p* < 0.01) and correlation analysis also showed a positive relationship between depression and death anxiety (*r* = 0.77, *p* < 0.01), depression and anxiety (*r* = 0.83, *p* < 0.01), and anxiety with death anxiety (*r* = 0.85, *p* < 0.01).

**Table 3:** Bivariate correlations for variables

Variables	Anxiety	Self-compassion	Death anxiety
Depression	0.83**^a^	− 0.48**	0.77**
Anxiety	–	− 0.53**	0.85**
Self-compassion		–	− 0.46**
Death anxiety			–

^a^**p < 0.01

Table 4 indicates that all paths were significant *p* < 0.01. Bootstrapping analyses revealed significant indirect effects of psychological well-being (depression and anxiety) on death anxiety via self-compassion. In the model, psychological well-being outcomes (i.e., depression and anxiety) were put in as the independent variable, self-compassion as the mediator, and death anxiety as the dependent variable. One meditation analysis was conducted with 210 participants in order to examine whether self-compassion should be considered as a mediator for the association among depression and anxiety with death anxiety. The overall model explained 75.4% of variance in death anxiety, *F* (3, 206) = 102.43, *p* < 0.001). As predicted, there was a significant indirect path of depression on death anxiety (*β* = 0.065, *SE *= 0.35, *p*  < 0.03, 95% *CI* [*LL *= − 0.0083, *UL*: − 0.1654]) and anxiety on death anxiety (*β* = 0.089, *SE *= 0.09, *p* < 0.04, 95% *CI* [*LL *= − 0.0247, *UL*: − 0.1987]) via self-compassion because the bootstrapped confidence interval does not go through zero. That is, participants who had less depression and anxiety scored higher in self-compassion, that partly considered for the positive associations among depression and anxiety with death anxiety.

**Table 4:** The direct and indirect effects of variables

Direct paths	β	SE	t	*p*
Depression → death anxiety	0.28	0.136	4.19	< 0.001
Depression → self-compassion	− 0.36	0.296	8.67	< 0.001
Anxiety → death anxiety	0.30	0.034	7.10	< 0.001
Anxiety → self-compassion	− 0.32	0.016	5.12	< 0.001
Self-compassion → death anxiety	− 0.40	0.078	4.05	< 0.001

Indirect paths	β	SE	Lower limit	Upper limit
Depression → self-compassion → death anxiety	0.065	0.35	− 0.0083	− 0.1654
Anxiety → self-compassion → death anxiety	0.089	0.09	− 0.0247	− 0.1987

**Fig. 2:**
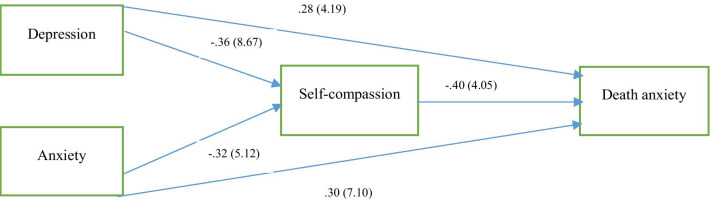
Path coefficients and t-values for the relationship between psychological outcomes (depression and anxiety) and death anxiety with mediating self-compassion

The hypothesized model with path coefficients and t-values for various paths is presented in Fig. 2. In the predicted model, psychological well-being outcomes (i.e., depression and anxiety) were conducted as the independent variable, self-compassion as the mediator, and death anxiety as the dependent variable. The results of the model indicate that all direct paths were significant *p* < 0.01.

Analysis of variance was used to make sure that the research variables were not influenced by age. For this purpose, the sample was first divided into three groups (18–33, 34–49 and 50–65). Results of analysis of variance indicated that death anxiety was not significantly different in the three age groups (F =  0.33, df_b _= 2, df_w _= 207, *p* = 0.79). Similar results were obtained for self-compassion (F = 1.07, df_b _= 2, df_w _= 207, p=.36), anxiety (F = 0.52, df_b _= 2, df_w_= 207, *p* = 0.66), and depression (F = 0.56, *p* = 0.64).

## Discussion

According to the results reported in Table 4, all paths were significant *p* < 0.01. As predicted, there was a significant indirect path of depression on death anxiety and anxiety on death anxiety via self-compassion, so women with breast cancer who widely practiced self-compassion had less depression and anxiety, as well as less fear of dying. That is, breast cancer survivors with less stress and anxiety have higher self-compassion scores, which partially mediated the relationship between depression and death anxiety. Recent research has shown that self-compassion, and its components, can be positively affected by interventions. According to a study by Kearney et al. on self-compassion and health issues, interventions that are rich in self-compassion can deliver better physical and psychological health benefits for people with breast cancer [38]. A meta-analysis of Randomized Control Trails (RCTs) of mental health demographics using compassion-based approaches found moderate impacts on anxiety (d = 0.49) and depression (d = 0.64) [39]. These results demonstrate that compassion plays a defensive function, which is extremely noteworthy in the present situation. There is a study among cancer patients indicating that higher self-compassion is related to a lower level of depression and psychopathology in cancer patients and increasing quality of life [29].

In another study, self-compassion was reported to be a strong predictor for mitigating social discomfort in women undergoing body changes because of breast cancer care [40]. Self-compassion encourages a person to really see their disease as a part of what it is to be human, to recognize that everyone struggles in some manner at some stage in their lives, and to care about and worry for oneself [41]. When dealt with an illness or disease, people with a high level of self-compassion are much more likely to be positive and take good care of their health [42]. Cancer patients may have concerns of mortality after a certain period of time has elapsed since their diagnosis, as well as treatment uncertainty. On top of that, death anxiety plays an important part in a variety of mental health problems during the COVID-19 outbreak [43].

In one clinical study, the amount of long-standing mental health diagnoses, number of prescriptions for mental health issues, (Depression, Anxiety and Stress Scale) DASS-21 depression, anxiety, and stress levels, as well as the symptom prevalence of 12 distinct diseases were shown to have substantial and favorable associations [44]. Upon controlling for neuroticism, these relationships remained important, suggesting that death anxiety has a significant position in psychopathology [43]. During the COVID-19 outbreak, cancer survivors may trigger concerns related to previous adverse health encounters, in particular being hospitalized during chemotherapy or being placed in neutropenia isolation [8]. Cancer sufferers are at an elevated risk of more serious infection and subsequent complications, according to two recent reports, particularly if surgery or chemotherapy is done within one month of Severe Acute Respiratory Syndrome Coronavirus 2 (SARSCov2), and a simple cancer diagnosis is linked to increased risk of death and/or ICU(Intensive Care Unit) entry [23, 45]. Cancer survivors may be concerned about future resource allocation due to ongoing questions about the availability of healthcare services, for example ventilators, which may be in short supply [46].

Providing a healthy atmosphere and efficient and compassionate care are more critical than ever during this epidemic, considering the well-being of cancer patients and families [47]. According to earlier studies, providing more self-compassion is linked to having less depression, anxiety, fatigue, and negative body image [40, 48]. To summarize, during periods of distress, like breast cancer, self-compassion can help to buffer the impact of stress [49]. Self-compassion skills may assist cancer survivors by facilitating self-care in the face of adversity [26]. It may be inferred that if breast cancer sufferers will be kind to themselves through stressful times of disease, embrace depression as a normal phase, and maintain a positive view of their health and recovery, they would be more able to cope with tension and challenges as they arise [50].

## Conclusion

The COVID-19 pandemic has triggered anxiety and depression, which could be far more serious in some patient populations, including cancer patients [47]. As hypothesized in this study, self-compassion has been found to statistically mediate the associations between psychological well-being (anxiety and depression) and death anxiety. This suggests that people with less stress and anxiety had better self-compassion scores, which may account for the relationship between depression and death anxiety. To enhance the well-being of cancer patients, it is important to create comfortable condition and best supportive care during the COVID-19 pandemic. There are effective self-compassion based therapies for depression and anxiety, and one study of a small-scale intervention that diminished cancer recurrence fear has already been released [51]. It would also be critical to identify the conditions that modify or promote intervention success, such as patients’ basic concerns and psychological characteristics [26].

### Limitations

There were some limitations in this study. The first, this study used convenient samples, which may limit the generalizability of current findings. Second, cross-sectional designs have two flaws: they do not provide inferences on the order or cause of mediational interactions, and they can be skewed by popular prejudice. Common method bias (response biases such as halo effects, social desirability, acquiescence, leniency effects, or yea-and nay-saying) [52] likely explains a portion of variance shared between all variables. Third, this study was done in the Zanjan Province, so further analysis in other regions of Iran is needed to expand the generalizability of the current findings to all Iranian breast cancer survivors in different parts of the country. Finally, this research relied solely on self-report tests, which are prone to bias (for example, social desirability) and pose questions about shared process variance [53].

### Clinical and research implications

First, during a coronavirus contagion, not just the psychological symptoms of cancer, but also the enhanced social distancing and shielding steps many patients experience, should be addressed. Second, during the COVID-19 outbreak, elevated rates of anxiety and depression were found in women with breast cancer, indicating that special attention should be given to their psychological status [54]. Finally, self-compassion strategies for women with breast cancer are not adequately used despite the strong evidence that such strategies are effective in improving the mental health and health outcomes of patients [55]. Moreover, prospective research should examine the effect of such treatments on these patients. We hope that these implications will assist physicians in providing the best possible treatment for their patients as the COVID-19 pandemic unfolds.

## Data Availability

The dataset of this study would be available from corresponding author on reasonable request.

## Abbreviations

COVID-19: Coronavirus Disease 2019
PCR: Polymerase chain reaction
RCTs: Randomized control trails
SARS Cov2: Severe acute respiratory syndrome Coronavirus2
ICU: Intensive care unit
SEM: Structural equation modeling
DASS-21: Depression, Anxiety and stress scale
